# Supplementary material for: Antibodies targeting a conserved cryptic epitope at the influenza hemagglutinin head-stem interface via distinct binding modes
Source: bioRxiv. 2026 Oct 3:2026.09.24.754278. Preprint. [Version 2] doi: 10.64898/2026.09.24.754278 (PMC13648409; doi:10.64898/2026.09.24.754278)
Supplement: Supplementary file 1 [file media-1.pdf]

Table S1. Cryo-EM data collection, refinement and validation statistics.

	01.z.01 Fab +<br>Dar21 HA head +<br>FluA-20 Fab +<br>ADI-85647 Fab<br>(EMBD-XXXXX)<br>(PDB XXXX)	56.e.01 Fab +<br>Dar21 HA head +<br>FluA-20 Fab +<br>ADI-85647 Fab<br>(EMBD-XXXXX)
**Data collection and processing**		
Magnification	81,000	150,000
Voltage (kV)	300	200
Electron exposure (e^-/Å^2)	57.35	60.00
Defocus range (μm)	-0.6 – 2.0	-0.3 – 3.0
Pixel size (Å)	0.529	0.96
Symmetry imposed	C1	C1
Initial particle images (no.)	407,616	71,910
Final particle images (no.)	103,663	4,903
Map resolution (Å)	3.38	8.77
FSC threshold 0.143		
Map postprocessing	DeepEMhancer	N/A
**Refinement**		
Initial model used (PDB code)	N/A	N/A
Model composition		
Non-hydrogen atoms	3,998	
Protein residues	517	
Root-mean-square deviations		
Bond lengths (Å)	0.005	
Bond angles (°)	0.824	
Validation		
MolProbity score	1.99	
Clashscore	9.63	
Poor rotamers (%)	2.01	
Ramachandran plot		
Favored (%)	96.28	
Allowed (%)	3.52	
Disallowed (%)	0.20	

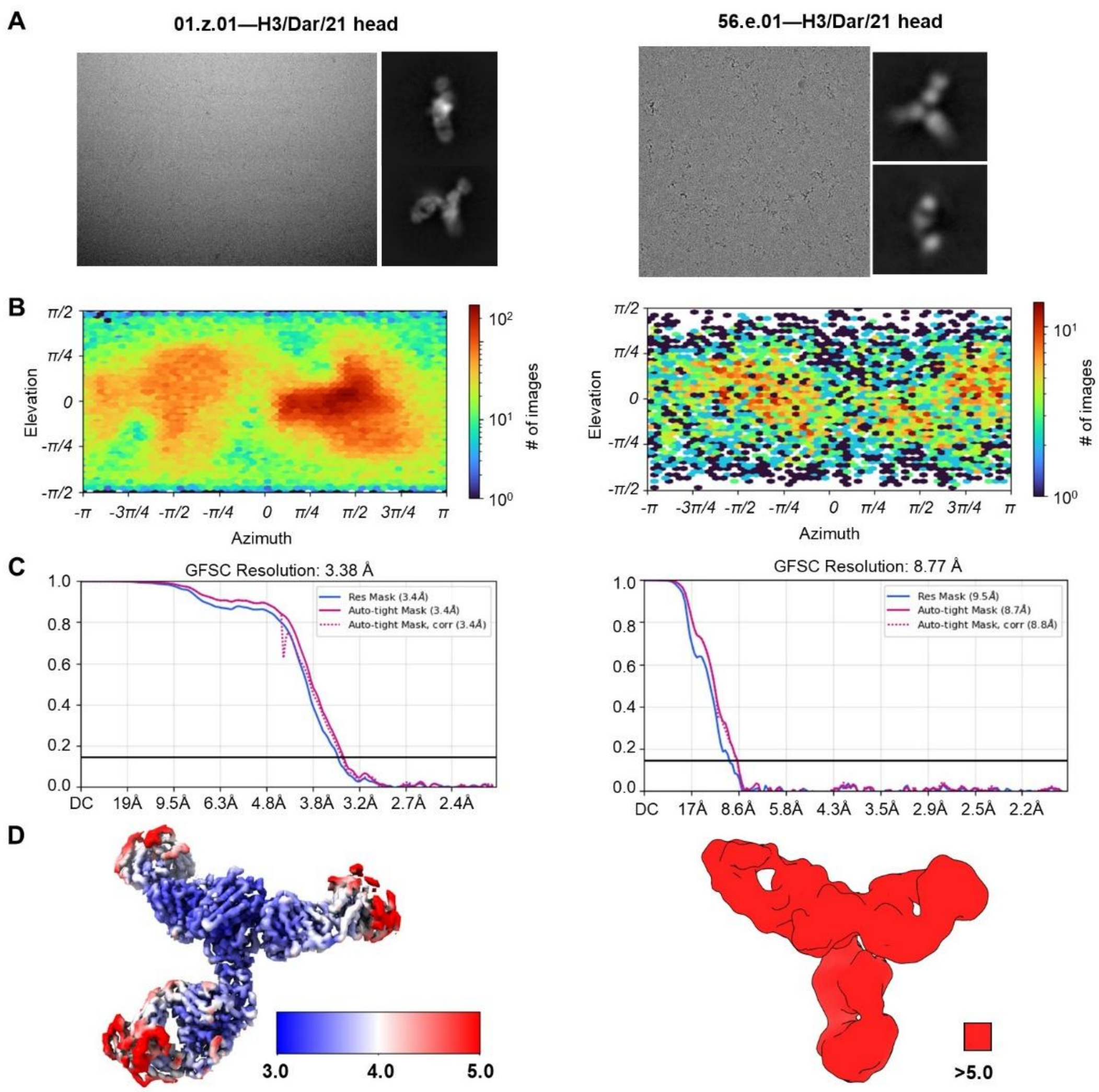

Figure S1: Cryo-EM data processing of Fab-HA complexes

CryoSPARC[1] data processing is shown for the 01.z.01 Fab + A/Darwin/9/2021(H3N2) HA head construct (left) and the 56.e.01 Fab + A/Darwin/9/2021(H3N2) HA head construct (right).

(A) Representative micrographs and 2D class averages for selected particles. 01.z.01 + H3 HA: 3820 micrographs, 407,616 particles picked, 103,663 particles selected to build 3D map. 56.e.01 + H3 HA: 290 micrographs, 71,910 particles picked, 4,903 particles selected to build 3D map.

(B) Viewing direction distribution of selected particles used to build each map.

(C) Gold-standard Fourier shell correlation curves for each map, with the 0.143 cutoff marked by a horizontal black line.

(D) Local resolution analysis of the respective complex maps.

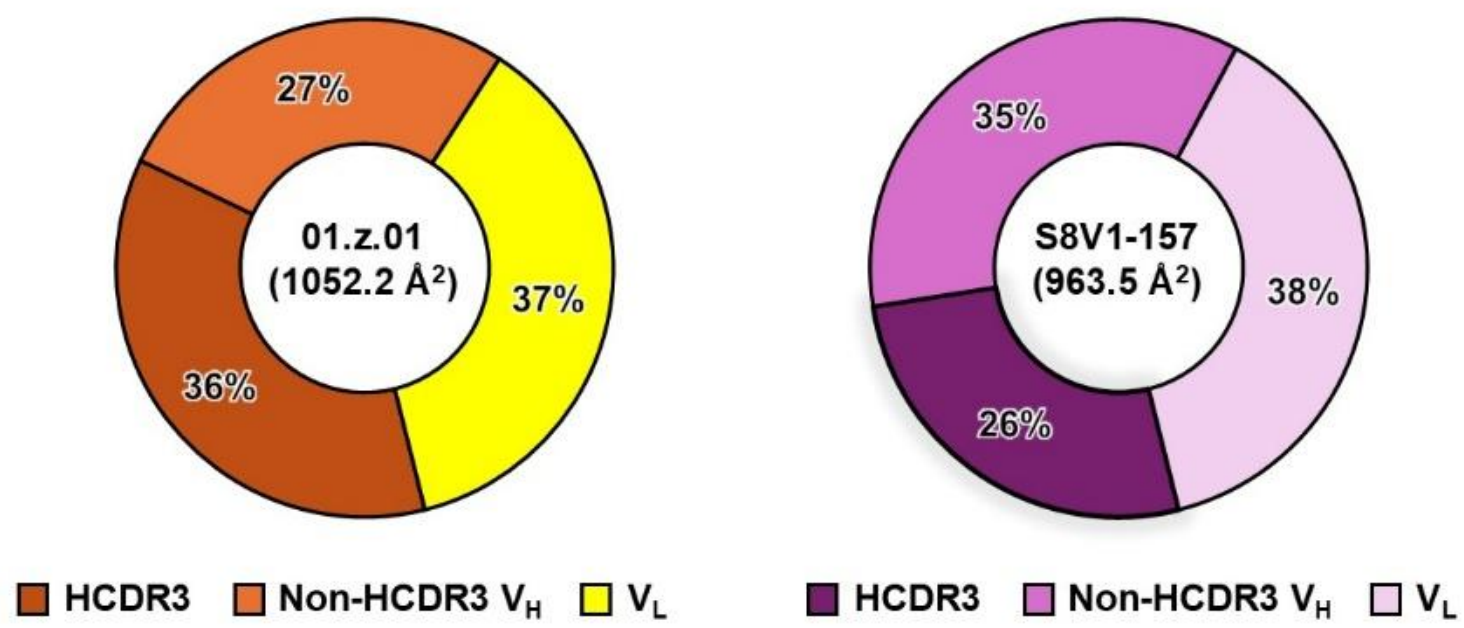

Figure S2. Buried surface area (BSA) analysis for head-stem interface antibodies

The paratope BSA for 01.z.01 is reported (left) as a fraction of the HCDR3 (dark orange), non-HCDR3 V_H (light orange), and V_L (yellow). The paratope BSA is similarly reported for S8V1-157 (PDB 8US0,[2] right) as a fraction of the HCDR3 (purple), non-HCDR3 V_H (orchid), and V_L (pink). Paratope BSA was calculated by PDBePISA[3].

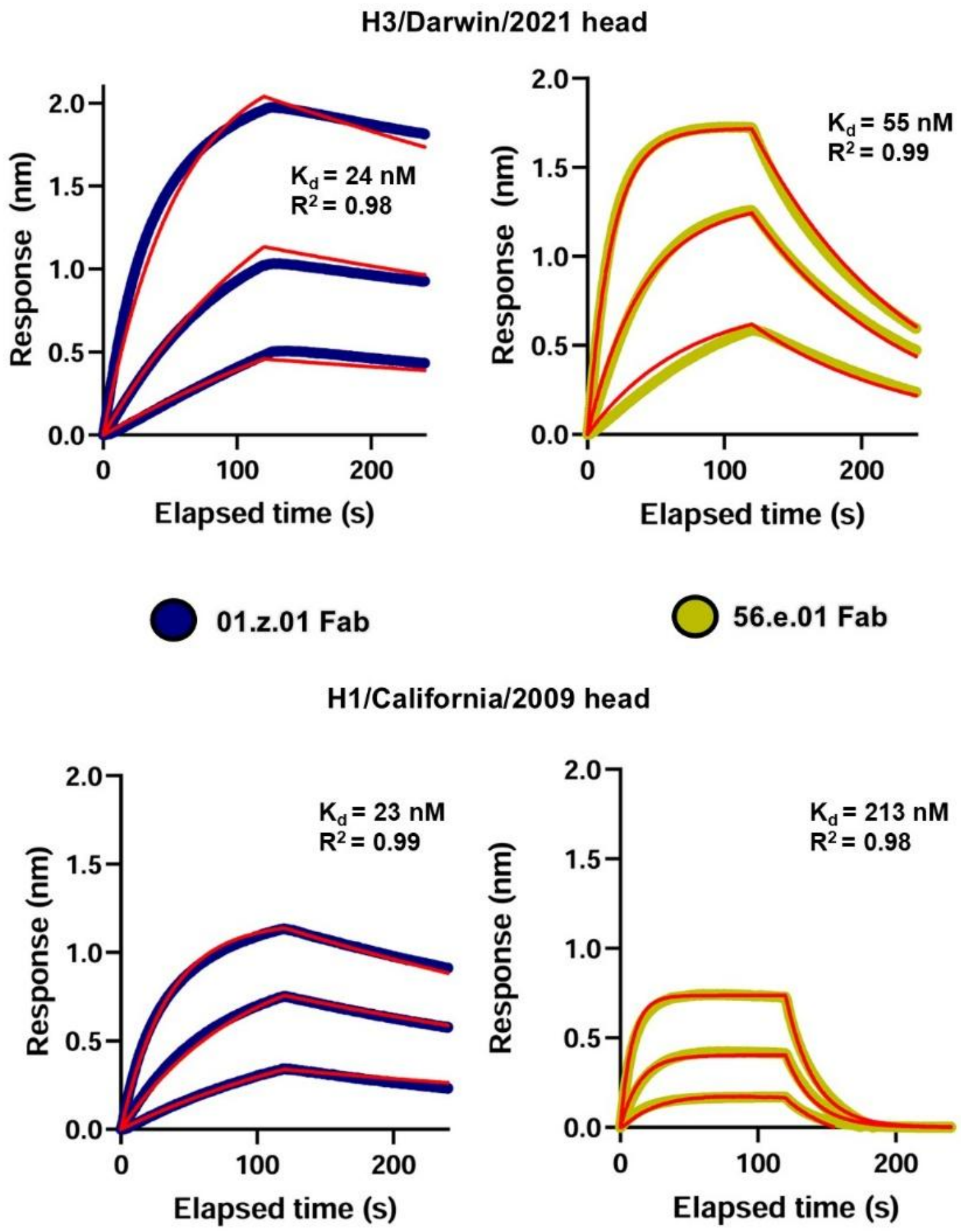

Figure S3. Kinetic profiling of head-stem interface Fab binding to HA head domain

Biolayer interferometry (BLI) data were collected at three Fab concentrations (300 nM, 100 nM, and 33 nM) on either immobilized A/Darwin/9/2021 (H3N2) HA1 (top) or A/California/04/2009 (H1N1) HA1 (bottom). Binding curves are shown for 01.z.01 Fab (left, dark blue) and 56.e.01 Fab (right, yellow). Curve fitting using a 1:1 binding model (red lines) was used to calculate dissociation constants (K_d).

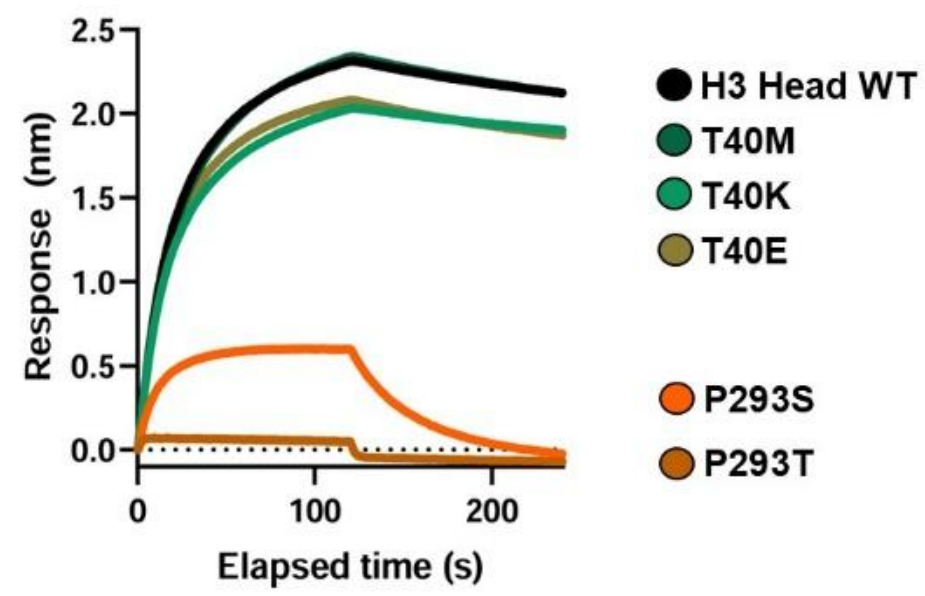

Figure S4. Binding analysis of S8V1-157 on H3 head mutants

Biolayer interferometry binding curves for 500 nM S8V1-157 Fab[2] against a panel of immobilized A/Darwin/9/2021 (H3N2) HA1 single-site variants.

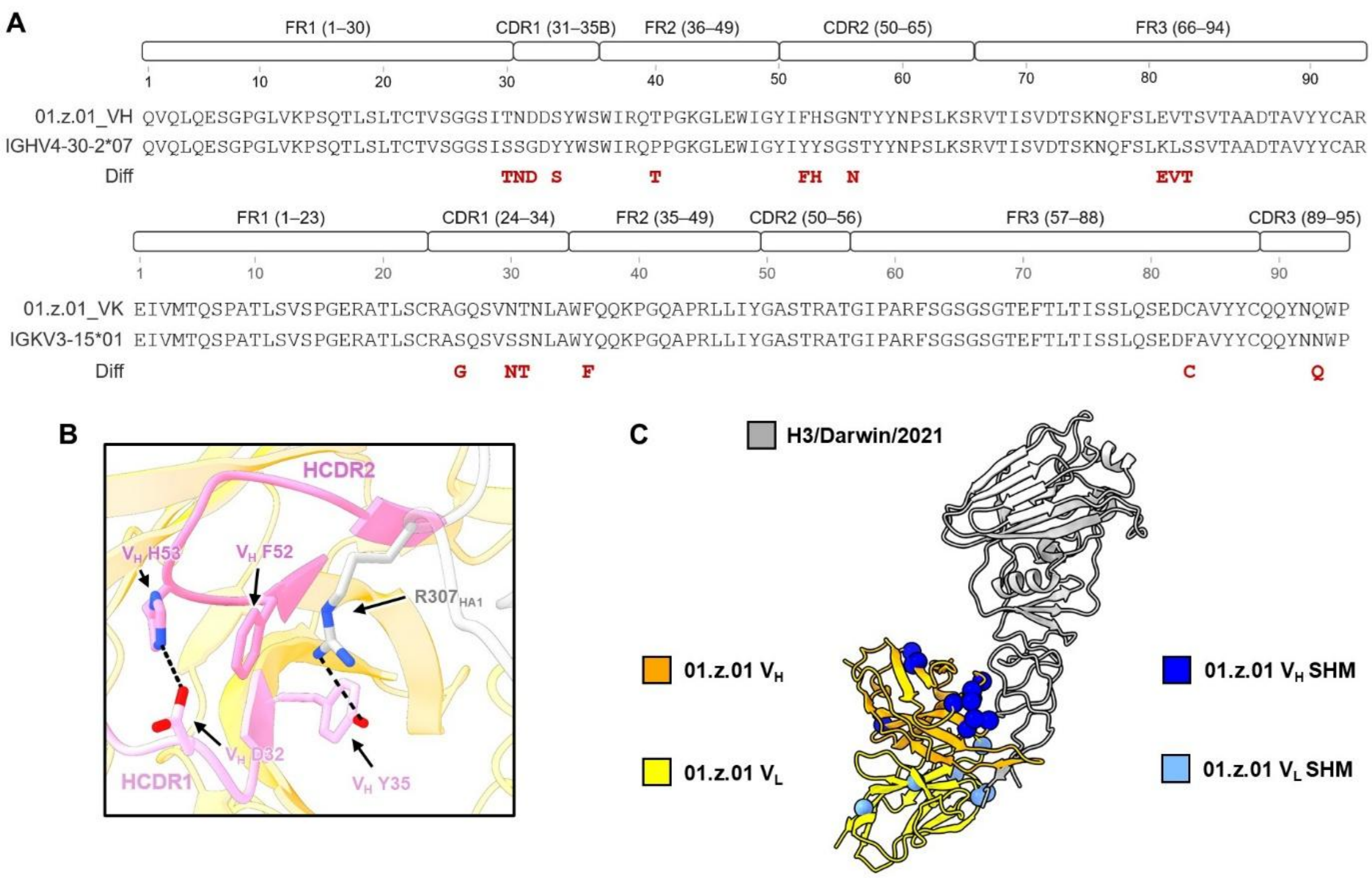

Figure S5. Germline gene alignment and somatic hypermutation analysis of 01.z.01

(A) Sequence alignment of 01.z.01 V_H (top) and V_L (bottom) with their respective inferred germline genes. Kabat numbering is employed. Locations of somatic hypermutations (SHMs) are shown in red.

(B) Molecular depiction of the involvement of residues arising from 01.z.01 SHM with the highly conserved $R307_{HA1}$. For clarity, the 01.z.01 HCDR1 (light pink) and HCDR2 (pink) are labeled.

(C) Ribbon diagram of 01.z.01 scFv (V_H: orange, V_L: yellow) in complex with A/Darwin/9/2021 (H3N2) HA1 (light gray). C_α atoms from residues arising from 01.z.01 SHM are depicted as spheres (V_H SHM: dark blue, V_L SHM: light blue).

Supplemental references

1. Punjani, A., Rubinstein, J. L., Fleet, D. J. & Brubaker, M. A. cryoSPARC: algorithms for rapid unsupervised cryo-EM structure determination. *Nat. Methods* **14**, 290–296 (2017).
2. Simmons, H. C. *et al.* A protective and broadly binding antibody class engages the influenza virus hemagglutinin head at its stem interface. *mBio* **16**, e00892-25 (2025).
3. Krissinel, E. & Henrick, K. Inference of macromolecular assemblies from crystalline state. *J. Mol. Biol.* **372**, 774–797 (2007).
